# Social support and medication adherence among adult myasthenia gravis patients in China: the mediating role of mental health and self-efficacy

**DOI:** 10.1186/s13023-024-03145-6

**Published:** 2024-04-04

**Authors:** Jiazhou Yu, Luyao Xie, Shanquan Chen, Zhilan Fang, Liling Zhu, Huanyu Zhang, Richard H. Xu, Huan Yang, Dong Dong

**Affiliations:** 1 The Jockey Club School of Public Health and Primary Care, The Chinese University of Hong Kong, Hong Kong SAR, China; 2 Department of Population Health, Faculty of Epidemiology and Population Health, London School of Hygiene and Tropical Medicine, London, UK; 3 Shenzhen Research Institute, The Chinese University of Hong Kong, Shenzhen, China; 4 Clinical Big Data Research Center, The Seventh Affiliated Hospital, Sun Yat-sen University, Shenzhen, China; 5 Department of Rehabilitation Sciences, The Hong Kong Polytechnic University, Hong Kong SAR, China; 6 Department of Neurology, Xiangya Hospital, Central South University, Changsha, China

**Keywords:** Myasthenia gravis, Medication adherence, Social support, Mental health, Self-efficacy

## Abstract

**Background:**

Myasthenia gravis (MG), a rare chronic neuromuscular disorder, is characterized by progressive physical decline and requires long-term pharmacological treatment. Due to the decline of physical and social abilities, MG patients are in great need of social support, including tangible and emotional support. This study aims to examine the association between social support and medication adherence and the possible mediating effects of mental health and self-efficacy among MG patients.

**Methods:**

A cross-sectional analysis of a nationwide MG registry was conducted on 865 patients under oral medication treatment in China between June and July 2022. Validated scales were used to measure the respondent’s mental distress (Four-item Patient Health Questionnaire), social support (Modified Medical Outcomes Study Social Support Scale), self-efficacy for medication use (Self-efficacy for Appropriate Medication Use Scale), and medication adherence (Morisky Medication Adherence Scale, MMAS).

**Results:**

The association between social support and medication adherence and possible mediating effects of mental distress and self-efficacy were tested by structural equation model, with significant demographic and disease-related factors adjusted. The respondents showed a very low level of medication adherence (71.2% poor adherence; 1.4% high adherence; mean MMAS = 4.65). The level of social support was positively associated with medication adherence, and such association was fully mediated by two indirect pathways: through self-efficacy (β = 0.07, proportion mediated = 63.8%); and through mental distress and then self-efficacy (β = 0.01, proportion mediated = 6.7%).

**Conclusion:**

Provision of social support and interventions on mental health with emphasis on improving self-efficacy for medication use may effectively improve medication adherence among MG patients.

**Supplementary Information:**

The online version contains supplementary material available at 10.1186/s13023-024-03145-6.

## Background

Myasthenia gravis (MG) is a rare chronic neuromuscular disorder characterized by progressive physical decline and requires long-term pharmacological treatment. Globally, the incidence of MG is approximately 1.7 to 21.3 per million person-years and the prevalence is estimated to be 15 to 179 per million persons [1]. The symptoms of MG may include weakness of eye muscles, difficulty in swallowing and breathing, and weakness in the arms, legs, and other muscles [2]. Due to the chronic, fluctuating muscle weakness, MG patients may present impaired physical functioning as well as a wide range of psychological and social disabilities. There is evidence that MG patients may suffer from fear, social anxiety, social avoidance, and depression [3–5], which could affect their health behaviors and quality of life [6, 7].

There is no cure for MG to date, but the prognosis of MG patients is generally good due to advances in pharmacological treatment, plasmapheresis, thymectomy, and critical illness management [8]. Compared to other chronic conditions like hypertension and diabetes, pharmacological treatment for MG patients, which is recommended as first-line treatment [8], often involves complex regimens, for example, concurrent use of multiple medications for treating neuromuscular symptoms (e.g. acetylcholinesterase inhibitor and immunosuppressant) and supplementary medications for treating comorbidities and drug side effects (e.g. nausea, weight gain, and infection resulted from long-term use of corticosteroids and azathioprine [9]). It has been reported that non-adherence is common among MG patients (38–65%), contributing to elevated risk of relapses, hospitalization, and crisis in patients as well as the increased cost of healthcare [10–13].

Previous studies on chronic diseases have demonstrated that medication non-adherence can be contributed by a confluence of factors, among which inadequate social support is underlined as a key variable, particularly when the treatment regimen is complex [14–17]. However, to date, no empirical studies have explored the role of social support among MG patients. Social support is defined as the social resources that individuals perceived to be available or that are actually provided to them [18]. Recent research has identified social support as a protective factor for physical and mental health [19].

While the mechanism of how social support affects medication adherence is not completely clear, some studies have highlighted the possible psychological pathway of the relationship. For example, stress-buffering model of support hypothesized that social support reduces stress perception and weakens the link between stress and adverse outcomes [18]. Previous studies have found that higher levels of support are associated with lower exposure and perception of stress and lower level of depression [20, 21]. In turn, mental distress is associated with decreased medication adherence as it may affect patients’ desire and ability to adhere to advised medication regimen [22, 23].

There is also strong evidence that social support may foster the sense of control over life and lead to a greater feeling of self-efficacy [24, 25]. Self-efficacy, defined as the belief or confidence that one can perform a specific required action successfully to achieve a desired outcome, has been noted as one of the most predominant predictors of health-promoting behavior and an important aspect of disease management [26–28]. Self-efficacy for appropriate medication use refers to the ability to take medications as advised, even under difficult or uncertain circumstances [29]. Studies on patients with chronic conditions have identified the mediating roles of psychological symptoms (depression and anxiety) and self-efficacy in the association between social support and medication adherence [30, 31]. Further, self-efficacy was also found as a mediator in the association between mental health and medication adherence [32, 33].

**Fig. 1:**
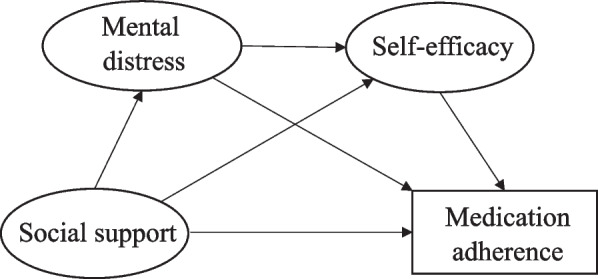
Proposed model of relationships between social support, anxiety/depression, medication self-efficacy, and medication adherence

Based on the existing literature mentioned above, social support, mental health, self-efficacy, and medication compliance are related and share complex relationships. Understanding the psychosocial profiles of MG patients, identifying their links with medication adherence, and clarifying the underlying mechanism may assist in devising targeted interventions for improving disease management and health outcomes of patients. This study aims to examine: (1) the levels of medication adherence, social support, mental distress, and self-efficacy; (2) the association between social support and medication adherence and the mediating role of mental health and self-efficacy in a large sample of MG patients. We hypothesized that social support would be associated with medication adherence, and that MG patients with higher social support would have lower level of mental distress and higher level of self-efficacy, which leads to better medication adherence (Fig. 1).

## Methods

### Study design and data collection

A web-based, cross-sectional survey was conducted on patients with MG between June and July 2022 in China. A pilot-tested questionnaire was distributed by Beijing Aili Myasthenia Gravis Care Center, the largest national MG patient organization in China, to its registered patients through social network platform. The patient registry system was established in May 2018, and more than 7,000 MG patients across China has voluntarily registered up to March 2023. All members were required to submit a proof of diagnosis for registration, which was subsequently verified by the organization. Invitations for the current survey were sent directly to approximately 3,000 registered MG patients who had participated in either of the two routine registry surveys in 2018 and 2019, using the organization’s internal patient communication network. The questionnaire for the current study collected the respondent’s information on socio-demographic characteristics (age, sex, marital status, education attainment, employment, and household income), disease-related information (duration of disease, use of medications), social support, mental distress, self-efficacy for medication use, and medication adherence. An informed consent form was presented prior to the start of online survey, and participants would only be directed to the survey if they agreed to participate and clicked “next page” button. To reduce the possibility of incompletion, the survey allowed the participants to exit temporarily, with the progress automatically resumed when accessed again. After the respondents completed the survey, answers were uploaded to an online platform and were manually checked by three researchers. Missing values, extreme values, and unusual answers were manually verified by trained staff by confirming with the patient or caregiver. Completion time of the survey was also manually reviewed to ensure that all the responses were completed within a reasonable duration. A total of 1,020 completed questionnaires were collected. Adult MG patients with generalized MG and were under medication treatment at the time of survey were considered eligible to include in data analysis. Finally, a total of 865 MG patients were included. Ethical approval of this study was obtained from the sponsoring university of the study.

### Instruments and measurements

#### Medication adherence

Medication adherence was measured by the 8-item Morisky Medication Adherence Scale (MMAS), which evaluates non-adherent behavior to medical regimen under different circumstances [34]. Items 1–7 of MMAS are scored by binary options, with “yes” scored by 1 point and “no” scored by 0 point. Item 8 is scored by a 5-point Likert scale, with responses of “never”, “once in a while”, “sometimes”, “usually”, and “all the time” scored by 1, 0.75, 0.50, 0.25, and 0, respectively. The total score is calculated by summing the scores from all items, ranging from 0 to 8, with higher score indicating better medication adherence [35]. The level of medication adherence was considered high, medium, and low with the MMAS score of 8, 6 to < 8, and < 6, respectively [35]. MMAS is one of the most widely used scale for measuring medication adherence and has shown favorable psychometric properties in various populations and settings [36]. The Chinese version of MMAS has been validated among patients with myocardial infarction (Cronbach’s α = 0.77) [37] and epilepsy (Cronbach’s α = 0.56) [38]. The Cronbach’s α is 0.61 in the present study.

#### Social support

Social support was measured by eight-item Modified Medication Outcomes Study Social Support Scale (mMOS-SSS), which contains two dimensions: tangible support and emotional support [39, 40]. Four items measure tangible support (e.g. “Someone to help you if you were confined to bed”) and four items measure emotional support (e.g. “Someone who understands your problems”). Each item is scored by a 5-point Likert scale, ranging from none of the time (1 point) to all of the time (5 points). Score of the scale was calculated by averaging the scale items and transformed to a 0–100 scale, with higher scores indicating higher level of support. The Chinese version of mMOS-SSS was adapted from the Chinese version of MOS-SSS, which has been validated among patients with chronic conditions [41]. The mMOS-SSS is a validated scale with good internal consistency, with Cronbach’s α ranging from 0.88 to 0.93 [39]. The internal consistency in the present sample was comparable (Cronbach’s α = 0.91).

#### Mental distress

Mental distress was measured by the Four-item Patient Health Questionnaire (PHQ-4), which screens for symptoms of depression and anxiety [42]. The instrument evaluates the respondent’s mental health by asking the frequency of specific symptoms over the past two weeks. The first two items are related to anxiety (e.g. feeling nervous, anxious or on edge) and the last two items are related to depression (e.g. feeling down, depressed or hopeless). Each item is scored by a 4-point Likert scale, ranging from not at all (0 point) to nearly everyday (3 points). Given that the symptoms of depression and anxiety are highly correlated (r = 0.80), a sum score of all items was calculated to reflect mental health status, ranging from 0 to 12, with higher score indicating higher worse mental health status. The overall score is rated as normal (0–2), mild (3–5), moderate (6–8), or severe (9–12), and anxiety/depression is defined as score ≥ 3 [42]. The Chinese version of PHQ-4 used in this study was adapted from the validated Chinese version of PHQ-8 [43], and was found of good internal consistency in our sample (Cronbach’s α = 0.91).

#### Self-efficacy for medication use

Self-efficacy *of medication use* was assessed by the 13-item Self-efficacy for Appropriate Medication Use Scale (SEAMS), which measures the respondent’s level of confidence in taking medications correctly under different circumstances [29]. Each item is scored by a 3-point Likert scale, ranging from not confident (1 point) to very confident (3 points). The total score is calculated by summing the scores from all items, ranging from 13 to 39, with higher score indicating higher level of confidence. The Chinese version of SEAMS showed good reliability and internal consistency (Cronbach’s α ranged from 0.83 to 0.92) among stroke patients [44]. The internal consistency is also good in the present sample (Cronbach’s α = 0.94).

#### Covariates

Covariates considered for analysis included age (18–30, 31–50, > 50), sex, marital status (single, married, divorced/widowed), education attainment (high school or below, above high school), employment (employed, unemployed), household income [< ¥3,000, ¥3,000–5,000, ¥5,000–10,000, ¥10,000 or above, (CNY¥1 = USD$0.15)], duration of disease (measured by years from date of diagnosis to date of data collection), number of medications currently in use (including pyridostigmine, corticosteroids, azathioprine, mycophenolate mofetil, mycophenolic acid, tacrolimus, cyclosporine, cyclophosphamide, methotrexate, Traditional Chinese Medicine, and other medicines), and current use of corticosteroids (yes, no).

### Statistical analysis

The basic characteristics and levels of social support, mental health, self-efficacy, and medication adherence were described. The continuous variables were reported as mean and standard deviation (SD), and categorical variables were reported in number and percentage. The scores of scales measuring social support, mental health, self-efficacy, and medication adherence were also reported in median and interquartile ranges (IQR). Univariable and multivariable linear regression analyses were conducted to identify socio-demographic and disease-related factors associated with medication adherence. The multivariable model followed a backward stepwise approach for variable selection. The identified variables from the multivariable model were adjusted in the following mediation analysis. The above analyses were conducted using Stata 16.0 (Stata Corp LP, College Station, TX, USA).

The mediation analyses were performed using a two-stage procedure of structural equation model (SEM) on Mplus 8.0, with estimation method of maximum likelihood [45]. Confirmatory factor analysis (CFA) was first conducted to examine the adequacy of the measurement for social support, mental health, and self-efficacy, which were built as latent variables. Then, SEM was performed to examine the goodness of fit of the hypothesized model. The SEM measures the indirect effect between social support and medication adherence through three mediation pathways: (1) mental health only, (2) self-efficacy only, (3) serially through first mental health and then self-efficacy. Socio-demographic and disease-related variables that were significant in the linear regressions were also controlled in the SEM. The model fit was considered to be satisfactory when: absolute fit (χ2/*df*) < 5, root mean square error of approximation (RMSEA) < 0.08, comparative fit index (CFI) > 0.90, Tucker-Lewis Index (TLI) > 0.90 [46, 47]. Standardized path coefficients (β) were presented and the mediation effect was assessed using a bootstrapping approach (*n* = 2,000). Statistical significance was set at *p* = 0.05.

## Results

### Background characteristics

The background characteristics of participants are described in Table 1. A total of 238 (27.5%) males and 627 (72.5%) females were included in the analysis. The age of participants ranged between 19 and 79, and the mean age was 41.1 (SD 11.3) years. Epidemiological data showed that the occurrence of MG tends to differ by sex and age, with more females affected for age below 40 (with a female:male ratio of 3:1), roughly equally affected for age 40–50, and more males affected for age above 50 [48]. In our sample, the female:male ratio was 3.3, 1.8, and 0.8 for the age of MG onset < 40, 40–50, and > 50, respectively, largely consistent with the epidemiological pattern of MG. More than half (64.8%, *n* = 542) of participants were not employed. The duration of MG ranged from 0 to 51 years, and the mean duration was 10.8 (SD 7.9) years. Approximately 73.5% (*n* = 636) of patients were taking more than one medication, and more than half (58.6%, *n* = 507) were using corticosteroids drug. The different combinations of medication regimen are described in Table S1. The most common regimen was use of pyridostigmine only (16.0%) and combined use of pyridostigmine and corticosteroids (13.4%).

**Table 1:** Background characteristics of participants (*n* = 865)

Variable	*n* (%)
Age (years)	
18–30	139 (16.1%)
31–50	527 (61.0%)
> 50	199 (23.0%)
Sex	
Male	238 (27.5%)
Female	627 (72.5%)
Marital status	
Single	163 (18.8%)
Married	610 (70.5%)
Divorced/widowed	92 (10.6%)
Education	
High school or below	414 (47.9%)
Above high school	451 (52.1%)
Employment	
Employed at least part-time	295 (35.2%)
Not employed	542 (64.8%)
Household monthly income (CNY)	
< ¥3,000	218 (25.2%)
¥3,000–5,000	237 (27.4%)
¥5,000–10,000	232 (26.8%)
¥10,000 or above	178 (20.6%)
Disease duration (years since diagnosed)	
Mean (SD)	10.8 (7.9)
Number of medications currently in use^a^	
1	229 (26.5%)
2	296 (34.2%)
3	263 (30.4%)
4	74 (8.6%)
5 or above	3 (0.4%)
Current use of corticosteroids	507 (58.6%)

CNY¥1 = USD$0.15

^a^Medications include pyridostigmine, corticosteroids, azathioprine, mycophenolate mofetil, mycophenolic acid, tacrolimus, cyclosporine, cyclophosphamide, methotrexate, Traditional Chinese Medicine

### Medication adherence, social support, mental distress, and self-efficacy

The levels of different scales for measuring medication adherence, social support, mental distress, and medication self-efficacy are described in Table 2. The mean score of MMAS was 4.65 (SD 1.74) and median was 4.75 (IQR 3.50–6.00). Only 12 patients (1.4%) had high adherence to treatment; 27.4% (*n* = 237) of patients had medium level of adherence, and the majority (71.2%, *n* = 616) of patients were of low adherence. The mean and median score of mMOS-SSS was 52.51 (SD 23.20) and 50.00 (IQR 34.38–68.75), respectively. Overall, the level of tangible support (mean score 54.74, median score 56.25) received by participants was higher than that of emotional support (mean score 50.29, median score 50.00). The mean and median score of PHQ-4 was 4.74 (SD 3.27) and 4.00 (IQR 3.00–7.00), respectively, and the overall level of depression (mean score 2.48, median score 2.00) was slightly higher than the level of anxiety (mean score 2.26, median score 2.00). Among all participants, 13.8% (*n* = 119) were considered with severe mental distress, 18.7% (*n* = 162) with moderate distress, 42.8% (*n* = 370) with mild distress, and 24.7% (*n* = 214) were normal. Approximately one-third of participants showed symptoms of depression (37.3%, *n* = 323) and anxiety (31.2%, *n* = 270). The mean and median score of SEAMS was 27.63 (SD 6.34) and 26.00 (IQR 24.00–33.00). Medication self-efficacy was higher under difficult circumstances (mean score 15.32, median score 14.00) than under uncertain circumstances (mean score 12.31, median score 12.00). The score was lowest for the item “when they cause some side effects”, followed by “when you are not sure how to take the medicine” (Table S2).

**Table 2:** Mean (SD), median (IQR), and score range of predictor variables and medication adherence among the sample (*n* = 865)

Variable	Mean (SD)	95% CI	Median (IQR)	Possible Range	Observed Range
Medication Adherence (MMAS)	4.65 (1.74)	4.53–4.77	4.75 (3.50, 6.00)	0–8	0.25–8
Social Support (mMOS-SSS)	52.51 (23.20)	50.97–54.06	50.00 (34.38, 68.75)	0–100	0–100
Tangible support	54.74 (24.58)	53.10–56.38	56.25 (37.50, 75.00)	0–100	0–100
Emotional support	50.29 (24.07)	48.68–51.90	50.00 (31.25, 68.75)	0–100	0–100
Mental distress (PHQ-4)	4.74 (3.27)	4.52–4.96	4.00 (3.00, 7.00)	0–12	0–12
Anxiety	2.26 (1.76)	2.15–2.38	2.00 (1.00, 3.00)	0–6	0–6
Depression	2.48 (1.69)	2.36–2.59	2.00 (1.00, 4.00)	0–6	0–6
Self-efficacy for medication use (SEAMS)	27.63 (6.34)	27.19–28.07	26.00 (24.00, 33.00)	13–39	13–39
Under difficult circumstances	15.32 (3.71)	15.08–15.57	14.00 (13.00, 19.00)	7–21	7–21
Under uncertain circumstances	12.31 (3.19)	12.10–12.52	12.00 (10.00, 14.00)	6–18	6–18

### Socio-demographic and disease-related factors of medication adherence

The results of univariable linear regression (Table S3) showed that age (*p* < 0.001 for > 50 years), sex (*p* = 0.04), education (*p* = 0.01), household income (*p* = 0.01 for ¥5,000–10,000; *p* = 0.003 for ¥10,000 or above), and duration of disease (*p* = 0.001) were significantly associated with medication adherence. Number of medications currently in use (*p* = 0.34) and current use of corticosteroids (*p* = 0.57) were not significantly associated with medication adherence. The association between social support and medication adherence was significant (β = 0.01, *p* = 0.007) after controlling for age, education, and duration of disease, but became non-significant (β = 0.001, *p* = 0.60) after adding mental distress (β = -0.03, *p* = 0.11) and medication self-efficacy (β = 0.07, *p *< 0.001) in the model.

### Testing of the hypothesized model

The results of correlation analysis (Table S4) showed that medication adherence was significantly positively correlated with social support (*p* <  0.001), medication self-efficacy (*p* < 0.001), age (*p* < 0.001), and education (*p* = 0.01), and negatively correlated with mental distress (*p* < 0.001).

The measurement model was tested using CFA. The model showed good fit with the data (χ2/*df* = 4.60, CFI = 0.92, TLI = 0.91, RMSEA = 0.07). All observed variables were significantly loaded on the corresponding latent variables (*p* < 0.001). The factor loadings of the three latent variables (social support, mental distress, and self-efficacy) ranged from 0.63 to 0.92 (Table S5).

**Fig. 2:**
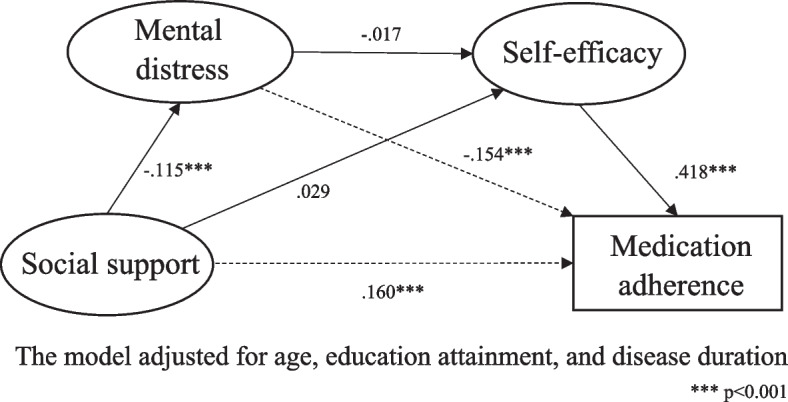
Structural model of relationships between social support, mental distress, medication self-efficacy, and medication adherence

The results of SEM suggest that the hypothesized model (Fig. 2) demonstrated a good fit after controlling for the significant background variables (χ2/df = 4.22, CFI = 0.91, TLI = 0.90, RMSEA = 0.06). Significant positive associations were found between social support and self-efficacy (β = 0.16, *p* < 0.001) and between self-efficacy and medication adherence (β = 0.42, *p* < 0.001). Significant negative associations were found between social support and mental distress (β = -0.12, *p* < 0.001) and between mental distress and self-efficacy = -0.15, *p* < 0.001). There was positive association between social support and medication adherence (β = 0.03, *p* = 0.38) and negative association between mental distress and medication adherence (β = -0.02, *p* = 0.45), but these associations were non-significant. The bootstrap results (Table 3) indicated that social support had an indirect effect (β = 0.08, *p* < 0.001) but non-significant direct effect (β = 0.03, *p* = 0.38) on medication adherence. Two mediating pathways were identified: (1) an indirect pathway through medication self-efficacy (β = 0.07, proportion mediated = 63.8%); (2) a serial indirect pathway through first mental distress, then self-efficacy (β = 0.01, proportion mediated = 6.7%). The mediating effect between social support and medication adherence through mental distress only was non-significant.

**Table 3:** Direct and indirect mediation effect in the proposed model

Path	Coefficient (95% CI)	Standard Error	*P*-value	Relative mediation effect
**Total effect**	0.11 (0.04, 0.17)	0.03	0.001	
**Direct effect**				
Social support➔ Medication adherence	0.03 (-0.04, 0.09)	0.03	0.38	
**Total indirect effect**	0.08 (0.05, 0.10)	0.01	< 0.001	
Specific indirect effect				
Social support ➔Mental distress➔ Medication adherence	0.002 (-0.003, 0.008)	0.003	0.48	——
Social support ➔Self-efficacy➔ Medication adherence	0.07 (0.04, 0.09)	0.01	< 0.001	63.8%
Social support ➔Mental distress➔ Self-efficacy➔ Medication adherence	0.01 (0.004, 0.01)	0.002	0.002	6.7%

## Discussion

Based on data of 865 MG patients from a nationwide patient registry, this study has revealed that MG patients in China showed a very low level of medication adherence, with only 1.4% highly adherent to their pharmacological treatment. We have also found that the level of social support is positively associated with medication adherence, and that such relationship is explained by indirect effects through mental health and self-efficacy. Specifically, patients with higher levels of social support are more likely to demonstrate better mental health and higher self-efficacy for medication use, which is a driver of higher medication adherence.

Medication adherence is a crucial component in effective treatment of MG patients. We found that the majority (71.2%) of the MG patients in China were poorly adherent to medication. The proportion of patients adherent to treatment (1.4%) is relatively low compared to findings from Brazil (44.8%) [13], Turkey (35.2%) [12], and Chile (61.5%) [49]. Nonetheless, it should be noted that these studies were conducted on small samples (sample size range 26–58) recruited from hospitals, which tend to overestimate treatment adherence in relative to the general MG patients in our study. The limited access to MG-specific healthcare services and suboptimal patient-clinician relationship could also contribute to the low level of medication adherence we identified [50]. In many places including China, knowledge and expertise in diagnosing and treating rare diseases are limited. Misdiagnosis of rare disease is common, which could lead to distrust in physicians and treatment regimen. As medical resources are usually concentrated in major cities, patients dispersed across the country may face difficulty in accessing resources, receiving timely treatment, and having regular follow-up in hospitals, which may contribute to inadequate communication with healthcare workers.

Our finding confirmed the hypothesis that the level of social support is positively associated with medication adherence, this in line with past studies on patients with other chronic diseases [16, 17]. Adding to the existing evidence, we have identified two pathways of mechanism in how social support may affect medication adherence indirectly and highlighted the key role of self-efficacy. First, MG patients with higher level of social support demonstrate higher self-efficacy, which contributes to higher medication adherence. Second, patients with adequate social support are less likely to develop depressive and anxiety symptoms, which in turn lead to elevated self-efficacy and eventually increases medication adherence. The crucial mechanism of self-efficacy has been previously demonstrated in studies on patients with other chronic conditions. Maeda et al*.* reported that self-efficacy fully mediated the relationship between social support and treatment adherence among patients with heart failure [51]. Evidence on hypertensive patients also showed that social support was related to self-management of disease via self-efficacy [52]. A study from US that explored the relationships between social support, depression/anxiety, and medication adherence identified a full mediation effect through depression/anxiety among persons living with HIV/AIDS [31]. Our study further revealed that the effect of mental distress on medication adherence may actually be through self-efficacy. Similar to patients with other chronic conditions, MG patients with high level of emotional and tangible support possibly have more external resources to coping with disease, higher self-esteem and sense of self-control, and therefore promote confidence in medication use [53]. Moreover, given the complex and dynamic nature of MG treatment regimen, the psychological factors, such as perceived support or good mood, in general, may not be able to affect the medication-taking behavior directly, but instead, through an indirect path by increasing the patients’ desire and ability to adhere to advised medication treatment.

The findings of this study yield some implications for practice. The key role of self-efficacy we identified suggests that social support and mental health interventions with emphasis on improving patients’ self-efficacy in medication use would be highly beneficial for medication adherence. We found that compared to patients with chronic conditions in general [44, 54], MG patients’ confidence in adhering to treatment were particularly impaired by concerns for drug side effects, uncertainty about medication schedule, and complexity of treatment regimen. Accordingly, interventions to enhance self-efficacy and thereby optimize medication adherence may consider support from family and friends that provide positive feedbacks on patients’ self-management, health education highlighting coping skills and knowledge about disease and treatment, and collaborative goal setting to strengthen patients’ ability and motivation to improve their self-efficacy and disease management. In clinical practice, instruction of medication use should be carefully communicated to patients, especially when multiple medications are involved. Meanwhile, healthcare workers should reinforce the importance of treatment adherence and dispel the patients’ fear for side effects. In addition to assessment and control of psychological symptoms which are commonly reported by MG patients, the clinical practice may incorporate mental health interventions that consider self-efficacy, for example, empowering the patients on disease management behaviors.

This study provided novel data on the level of medication adherence in a non-clinical sample of MG patients. Given the varied access to medical resources, our results, generated from real-world experience, may provide a more comprehensive picture of patient profile compared to past findings from clinical samples. The large sample size is another strength of this study, which increased the validity of findings. The study has contributed valuable data on self-management behavior and psychosocial aspects of MG patients and identified possible underlying mechanisms in the association between social support and medication adherence, which provided practical implications for management of MG.

There are several limitations to this study. First, the cross-sectional design precluded the establishment of causal relationship between different factors and medication adherence. Second, although the participants of this study were from 31 out of 33 provinces/municipals across China, it should be noted that the voluntary nature of patient registry and possible over-presentation of young patients resulted from web-based data collection approach may undermine the generalizability of our results to all MG patients. Third, the data collected by questionnaire were self-reported by participants and may be prone to information bias. However, key variables included in SEM were determined by validated scales, which increases the validity of results. Lastly, some variables that may also be associated with medication adherence were not evaluated, such as health literacy, attitude or belief toward illness and treatment, and patient-clinician relationship, which warrants future studies. As medication regimen is dynamic, data from longitudinal studies are needed to further confirm the findings of this study. Future studies may also consider assessing the effectiveness of different self-efficacy interventions on improving medication adherence and further exploring the mechanism between psychosocial factors and medication adherence among MG patients through qualitative approaches.

## Conclusions

It is imperative to improve medication adherence among MG patients. Provision of social support and interventions on mental health with emphasis on improving self-efficacy for medication use may facilitate improvement in medication adherence.

### Supplementary Information

**Supplementary Material 1.** 

## Data Availability

The data that support the findings of this study are owned by individual patients, and they have not given their permission for researchers to share their data. Data requestion can be made to the corresponding author on reasonable request via email.

## Abbreviations

CFA: Confirmatory factor analysis
CFI: Comparative fit index
MG: Myasthenia gravis
MMAS: Morisky Medication Adherence Scale
mMOS-SSS: Modified Medication Outcomes Study Social Support Scale
PHQ-4: Four-item Patient Health Questionnaire
RMSEA: Root mean square error of approximation
SEAMS: Self-efficacy for Appropriate Medication Use Scale
SEM: Structural equation model
TLI: Tucker-Lewis Index

## References

[1] Carr AS, Cardwell CR, McCarron PO (2010). A systematic review of population based epidemiological studies in Myasthenia Gravis. BMC Neurol.

[2] Gilhus NE (2016). Myasthenia Gravis. N Engl J Med.

[3] Richards HS, Jenkinson E, Rumsey N (2014). The Psychosocial Impact of Ptosis as a Symptom of Myasthenia Gravis: A Qualitative Study. Orbit.

[4] Chen YT, Shih FJ, Hayter M (2013). Experiences of Living With Myasthenia Gravis: A Qualitative Study With Taiwanese People. J Neurosci Nurs.

[5] Fisher J, Parkinson K, Kothari MJ (2003). Self-reported Depressive Symptoms in Myasthenia Gravis. J Clin Neuromuscul Dis.

[6] Leonardi M, Raggi A, Antozzi C (2010). The relationship between health, disability and quality of life in Myasthenia Gravis: results from an Italian study. J Neurol.

[7] Raggi A, Leonardi M, Antozzi C (2010). Concordance between severity of disease, disability and health-related quality of life in Myasthenia gravis. Neurol Sci.

[8] Romi F, Gilhus NE, Aarli JA (2005). Myasthenia gravis: clinical, immunological, and therapeutic advances. Acta Neurol Scand.

[9] Sanders DB, Evoli A (2010). Immunosuppressive therapies in myasthenia gravis. Autoimmunity.

[10] Murthy JM, Meena AK, Chowdary GV (2005). Myasthenic crisis: Clinical features, complications and mortality. Neurol India.

[11] Gummi RR, Kukulka NA, Deroche CB (2019). Factors associated with acute exacerbations of myasthenia gravis. Muscle Nerve.

[12] Aşiret G, Kapucu S, Kaymaz TT, et al. Psychosocial Adjustment and Adherence to Medication in Patients with Myasthenia Gravis. *GMJ*; 32. Epub ahead of print 1 July 2021. 10.12996/gmj.2021.85.

[13] Vitturi BK, Pellegrinelli A, Valerio BCO (2020). Medication adherence in patients with myasthenia gravis in Brazil: a cross-sectional study. Acta Neurol Belg.

[14] Strom JL, Egede LE (2012). The Impact of Social Support on Outcomes in Adult Patients with Type 2 Diabetes: A Systematic Review. Curr Diab Rep.

[15] Stopford R, Winkley K, Ismail K (2013). Social support and glycemic control in type 2 diabetes: A systematic review of observational studies. Patient Educ Couns.

[16] Shahin W, Kennedy GA, Stupans I (2021). The association between social support and medication adherence in patients with hypertension: A systematic review. Pharm Pract (Granada).

[17] Scheurer D, Choudhry N, Swanton KA (2012). Association Between Different Types of Social Support and Medication Adherence. Am J Manag Care.

[18] Cohen S (2004). Social Relationships and Health. Am Psychol.

[19] DiMatteo MR (2004). Social Support and Patient Adherence to Medical Treatment: A Meta-Analysis. Health Psychol.

[20] Russell DW, Cutrona CE (1991). Social support, stress, and depressive symptoms among the elderly: test of a process model. Psychol Aging.

[21] Cutrona CE, Russell DW. Type of social support and specific stress: Toward a theory of optimal matching. In: Sarason BR, Sarason IG, Pierce GR, editors. Wiley series on personality processes. Social support: An interactional view; 1990. p. 319–66.

[22] Bautista LE, Vera-Cala LM, Colombo C (2012). Symptoms of depression and anxiety and adherence to antihypertensive medication. Am J Hypertens.

[23] Grenard JL, Munjas BA, Adams JL (2011). Depression and medication adherence in the treatment of chronic diseases in the United States: a meta-analysis. J Gen Intern Med.

[24] Thoits PA (2011). Mechanisms Linking Social Ties and Support to Physical and Mental Health. J Health Soc Behav.

[25] Atienza AA, Collins R, King AC (2001). The mediating effects of situational control on social support and mood following a stressor: a prospective study of dementia caregivers in their natural environments. J Gerontol B Psychol Sci Soc Sci.

[26] Bodenheimer T, Lorig K, Holman H (2002). Patient self-management of chronic disease in primary care. JAMA.

[27] Bandura A (1986). Social foundations of thought and action: A social cognitive theory.

[28] Strecher VJ, DeVellis BM, Becker MH (1986). The role of self-efficacy in achieving health behavior change. Health Educ Q.

[29] Risser J, Jacobson TA, Kripalani S (2007). Development and Psychometric Evaluation of the Self-Efficacy for Appropriate Medication Use Scale (SEAMS) in Low-Literacy Patients With Chronic Disease. J Nurs Meas.

[30] Simoni JM, Frick PA, Lockhart D (2002). Mediators of Social Support and Antiretroviral Adherence Among an Indigent Population in New York City. AIDS Patient Care STDS.

[31] Huynh AK, Kinsler JJ, Cunningham WE (2013). The role of mental health in mediating the relationship between social support and optimal ART adherence. AIDS Care.

[32] Schoenthaler A, Ogedegbe G, Allegrante JP (2009). Self-efficacy mediates the relationship between depressive symptoms and medication adherence among hypertensive African Americans. Health Educ Behav.

[33] Son Y-J, Won MH (2017). Depression and medication adherence among older Korean patients with hypertension: Mediating role of self-efficacy. Int J Nurs Pract.

[34] Morisky DE, Green LW, Levine DM (1986). Concurrent and predictive validity of a self-reported measure of medication adherence. Med Care.

[35] Morisky DE, Ang A, Krousel-Wood M (2008). Predictive validity of a medication adherence measure in an outpatient setting. J Clin Hypertens (Greenwich).

[36] Kwan YH, Weng SD, Loh DHF (2020). Measurement Properties of Existing Patient-Reported Outcome Measures on Medication Adherence: Systematic Review. J Med Internet Res.

[37] Yan J, You L-M, Yang Q (2014). Translation and validation of a Chinese version of the 8-item Morisky medication adherence scale in myocardial infarction patients. J Eval Clin Pract.

[38] Yang A, Wang B, Zhu G (2014). Validation of Chinese version of the Morisky Medication Adherence Scale in patients with epilepsy. Seizure.

[39] Moser A, Stuck AE, Silliman RA (2012). The eight-item modified Medical Outcomes Study Social Support Survey: psychometric evaluation showed excellent performance. J Clin Epidemiol.

[40] Sherbourne CD, Stewart AL (1991). The MOS social support survey. Soc Sci Med.

[41] Wang W, Zheng X, He H-G (2013). Psychometric testing of the Chinese Mandarin version of the Medical Outcomes Study Social Support Survey in patients with coronary heart disease in mainland China. Qual Life Res.

[42] Kroenke K, Spitzer RL, Williams JBW (2009). An ultra-brief screening scale for anxiety and depression: the PHQ-4. Psychosomatics.

[43] Wang W, Bian Q, Zhao Y (2014). Reliability and validity of the Chinese version of the Patient Health Questionnaire (PHQ-9) in the general population. Gen Hosp Psychiatry.

[44] Dong X, Liu Y, Wang A (2016). Psychometric properties of the Chinese version of the Self-Efficacy for Appropriate Medication Use Scale in patients with stroke. Patient Prefer Adherence.

[45] Anderson JC, Gerbing DW. Structural equation modeling in practice: A review and recommended two-step approach. Psychol Bull. 1988;103(3):411–23.

[46] Browne MW, Cudeck R (1992). Alternative Ways of Assessing Model Fit. Sociological Methods & Research.

[47] Bentler PM (1990). Comparative fit indexes in structural models. Psychol Bull.

[48] Jayam Trouth A, Dabi A, Solieman N (2012). Myasthenia Gravis: A Review. Autoimmune Diseases.

[49] Idiaquez JF, Gonzalez S, Lasso-Penafiel J (2018). Pharmacological treatment compliance and a description of its associated factors in patients with myasthenia gravis. Rev Neurol.

[50] Kerse N (2004). Physician-Patient Relationship and Medication Compliance: A Primary Care Investigation. The Annals of Family Medicine.

[51] Maeda U, Shen B-J, Schwarz ER (2013). Self-Efficacy Mediates the Associations of Social Support and Depression with Treatment Adherence in Heart Failure Patients. IntJ Behav Med.

[52] Ding W, Li T, Su Q (2018). Integrating factors associated with hypertensive patients' self-management using structural equation model: a cross-sectional study in Guangdong. China PPA.

[53] Hogan BE, Linden W, Najarian B (2002). Social support interventions: Do they work?. Clin Psychol Rev.

[54] Alhazzani H, AlAmmari G, AlRajhi N (2021). Validation of an Arabic Version of the Self-Efficacy for Appropriate Medication Use Scale. IJERPH.

