# Supplementary material for: Social support and medication adherence among adult myasthenia gravis patients in China: the mediating role of mental health and self-efficacy
Source: Orphanet J Rare Dis. 2024 Apr 4;19:143. doi: 10.1186/s13023-024-03145-6 (PMC10993533; doi:10.1186/s13023-024-03145-6)
Supplement: Supplementary file 1 — Supplementary Material 1. [file 13023_2024_3145_MOESM1_ESM.docx]

**Additional File**

**Table S1.** Combinations of medications reported by respondents (n=865)

| Number of medications and combinations | n (%) |
| --- | --- |
| **1 medication** | **229 (26.5%)** |
| Pyridostigmine | 138 (16.0%) |
| Corticosteroids | 29 (3.4%) |
| Tacrolimus | 29 (3.4%) |
| TCM | 24 (2.8%) |
| Azathioprine | 7 (0.8%) |
| Cyclosporine | 2 (0.2%) |
| **2 medications** | **296 (34.2%)** |
| Pyridostigmine + corticosteroids | 116 (13.4%) |
| Pyridostigmine + TCM | 54 (6.2%) |
| Pyridostigmine + tacrolimus | 47 (5.4%) |
| Corticosteroids + tacrolimus | 22 (2.5%) |
| Corticosteroids + TCM | 20 (2.3%) |
| Corticosteroids + azathioprine | 10 (1.2%) |
| Pyridostigmine + azathioprine | 8 (0.9%) |
| Pyridostigmine + mycophenolic acid | 5 (0.6%) |
| TCM + azathioprine | 4 (0.5%) |
| TCM + tacrolimus | 3 (0.3%) |
| Corticosteroids + methotrexate | 2 (0.2%) |
| Pyridostigmine + cyclosporine | 1 (0.1%) |
| Corticosteroids + cyclosporine | 1 (0.1%) |
| Corticosteroids + cyclophosphamide | 1 (0.1%) |
| TCM + cyclophosphamide | 1 (0.1%) |
| Azathioprine + tacrolimus | 1 (0.1%) |
| **3 medications** | **263 (30.4%)** |
| Pyridostigmine + corticosteroids + TCM | 84 (9.7%) |
| Pyridostigmine + corticosteroids + tacrolimus | 82 (9.5%) |
| Pyridostigmine + corticosteroids + azathioprine | 40 (4.6%) |
| Pyridostigmine + TCM + tacrolimus | 22 (2.5%) |
| Corticosteroids + TCM + tacrolimus | 7 (0.8%) |
| Pyridostigmine + TCM + azathioprine | 7 (0.8%) |
| Pyridostigmine + corticosteroids + mycophenolic acid | 5 (0.6%) |
| Pyridostigmine + corticosteroids + cyclophosphamide | 4 (0.5%) |
| Corticosteroids + TCM + mycophenolic acid | 3 (0.3%) |
| Corticosteroids + TCM + cyclophosphamide | 2 (0.2%) |
| Pyridostigmine + corticosteroids + mycophenolate mofetil | 2 (0.2%) |
| Pyridostigmine + corticosteroids + cyclosporine | 1 (0.1%) |
| Corticosteroids + TCM + azathioprine | 1 (0.1%) |
| Pyridostigmine + TCM + mycophenolate mofetil | 1 (0.1%) |
| Pyridostigmine + TCM + methotrexate | 1 (0.1%) |
| Pyridostigmine + mycophenolic acid + mycophenolate mofetil | 1 (0.1%) |
| **4 medications** | **74 (8.6%)** |
| Pyridostigmine + corticosteroids + TCM + tacrolimus | 32 (3.7%) |
| Pyridostigmine + corticosteroids + TCM + azathioprine | 24 (2.8%) |
| Pyridostigmine + corticosteroids + TCM + mycophenolic acid | 5 (0.6%) |
| Pyridostigmine + corticosteroids + azathioprine + tacrolimus | 5 (0.6%) |
| Pyridostigmine + corticosteroids + TCM + cyclosporine | 2 (0.2%) |
| Pyridostigmine + corticosteroids + TCM + cyclophosphamide | 2 (0.2%) |
| Pyridostigmine + corticosteroids + mycophenolic acid + mycophenolate mofetil | 1 (0.1%) |
| Pyridostigmine + corticosteroids + mycophenolate mofetil + cyclosporine | 1 (0.1%) |
| Pyridostigmine + TCM + tacrolimus + cyclophosphamide | 1 (0.1%) |
| Pyridostigmine + mycophenolic acid + mycophenolate mofetil + cyclophosphamide | 1 (0.1%) |
| **5 medications** | **2 (0.2%)** |
| Pyridostigmine + corticosteroids + TCM + mycophenolic acid + tacrolimus | 1 (0.1%) |
| Pyridostigmine + corticosteroids + TCM + mycophenolic acid + mycophenolate mofetil | 1 (0.1%) |
| **6 medications** | **1 (0.1%)** |
| Pyridostigmine + corticosteroids + TCM + mycophenolic acid + tacrolimus + methotrexate | 1 (0.1%) |

TCM: traditional Chinese medicine.

**Table S2.** Mean (SD) of SEAM items

| Item | Mean (SD) |
| --- | --- |
| **Under difficult situations:** |  |
| 1- When your normal routine gets messed up? | 2.02 (0.69) |
| 2- When the schedule to take the medicine is not convenient? | 2.13 (0.68) |
| 3- When you have a busy day planned? | 2.16 (0.66) |
| 4- When you take several different medicines each day? | 2.19 (0.66) |
| 5- When you are away from home? | 2.20 (0.67) |
| 6- When you take medicines more than once a day | 2.30 (0.63) |
| 7- When no one reminds you to take the medicine? | 2.31 (0.64) |
| **Under uncertain situations:** |  |
| 8- When they cause some side effects? | 1.81 (0.71) |
| 9- When you are not sure how to take the medicine? | 2.01 (0.67) |
| 10- When you are feeling sick (like having a cold or the ﬂu)? | 2.04 (0.73) |
| 11-When you get a reﬁll of your old medicines and some of the pills look different than usual? | 2.07 (0.67) |
| 12- When you are not sure what time of the day to take your medicine? | 2.14 (0.65) |
| 13- When a doctor changes your medicines? | 2.24 (0.65) |

**Table S3.** Significant demographic and disease-related predictors of medication adherence

| Variable | Univariable regression |  |  | Multivariable regression | |  |
| --- | --- | --- | --- | --- | --- | --- |
|  |  | p-value | Without mental distress and self-efficacy | p-value | With mental distress and self-efficacy | p-value |
|  | *Coefficient (95% CI)* |  | *Coefficient (95% CI)* |  | *Coefficient (95% CI)* |  |
| Social support (mMMOS-SSS) | **0.01 (0.004, 0.01)** | **0.001** | **0.01 (0.002, 0.01)** | **0.007** | 0.001 (-0.004, 0.006) | 0.60 |
| Mental distress (PHQ-4) | **-0.09 (-0.12, -0.05)** | **<0.001** |  |  | -0.03 (-0.06, 0.07) | 0.11 |
| Medication self-efficacy (SEAMS) | **0.08 (0.07, 0.09)** | **<0.001** |  |  | **0.07 (0.05, 0.09)** | **<0.001** |
| Age |  |  |  |  |  |  |
| 18-30 | Ref. |  | Ref. |  | Ref. |  |
| 31-50 | 0.19 (-0.13, 0.52) | 0.24 | 0.32 (-0.05, 0.64) | 0.053 | 0.22 (-0.09, 0.53) | 0.16 |
| >50 | **0.71 (0.33, 1.08)** | **<0.001** | **0.83 (0.46, 1.21)** | **<0.001** | **0.63 (0.27, 0.99)** | **0.001** |
| Sex |  |  |  |  |  |  |
| Male | Ref. |  | —— |  | —— |  |
| Female | **-0.27 (-0.53, -0.01)** | **0.04** | —— |  | —— |  |
| Marital status |  |  |  |  |  |  |
| Single | Ref. |  | —— |  | —— |  |
| Married | 0.09 (-0.22, 0.39) | 0.58 | —— |  | —— |  |
| Divorced/widowed | 0.17 (-0.27, 0.62) | 0.44 | —— |  | —— |  |
| Education |  |  |  |  |  |  |
| High school or below | Ref. |  | Ref. |  | Ref. |  |
| Above high school | **0.30 (0.07, 0.53)** | **0.01** | **0.25 (0.01, 0.49)** | **0.04** | 0.05 (-0.17, 0.28) | 0.64 |
| Employment |  |  |  |  |  |  |
| Not employed | Ref. |  | —— |  | —— |  |
| Employed at least part-time | -0.17 (-0.41, 0.08) | 0.18 | —— |  | —— |  |
| Household monthly income (CNY) |  |  |  |  |  |  |
| <¥3,000 | Ref. |  | —— |  | —— |  |
| ¥3,000-5,000 | 0.29 (-0.03, 0.60) | 0.08 | —— |  | —— |  |
| ¥5,000-10,000 | **0.40 (0.08, 0.72)** | **0.01** | —— |  | —— |  |
| ¥10,000 or above | **0.53 (0.18, 0.87)** | **0.003** | —— |  | —— |  |
| Disease duration (years since diagnosed) | **-0.02 (-0.04, -0.01)** | **0.001** | **-0.03 (-0.04, -0.01)** | **<0.001** | **-0.03 (-0.04, -0.01)** | **<0.001** |
| Number of medications currently in use | 0.03 (-0.09, 0.15) | 0.34 | —— |  | —— |  |
| Current use of corticosteroids | 0.06 (-0.17, 0.30) | 0.57 | —— |  | —— |  |

CNY¥1=USD$0.15

Bold value indicates statistical significance

**Table S4.** Correlation between social support, anxiety and depression, medication self-efficacy, and medication adherence in coefficient (p-value).

| Variable | Medication adherence | Social Support | Mental distress | Medication self-efficacy | Age | Education | Disease duration |
| --- | --- | --- | --- | --- | --- | --- | --- |
| Medication adherence | 1 |  |  |  |  |  |  |
| Social Support | **0.12 (<0.001)** | 1 |  |  |  |  |  |
| Mental distress | **-0.16 (<0.001)** | **-0.22 (<0.001)** | 1 |  |  |  |  |
| Medication self-efficacy | **0.32 (<0.001)** | **0.27(<0.001)** | **-0.33(<0.001)** | 1 |  |  |  |
| Age | **0.16(<0.001)** | 0.05 (0.18) | -0.01 (0.79) | **0.13 (<0.001)** | 1 |  |  |
| Education | **0.09 (0.01)** | **0.17 (<0.001)** | **-0.18 (<0.001)** | **0.20 (<0.001)** | **-0.09 (<0.001)** | 1 |  |
| Disease duration | **-0.11 (<0.001)** | -0.05 (0.18) | 0.05 (0.15) | -0.03 (0.45) | **0.15 (<0.001)** | **-0.09 (0.006)** | 1 |

^Bold value indicates statistical significance^

**Table S5.** Unstandardized and standardized loadings for the measurement model

| Parameter estimates | | Unstandardized loading (SE) | Standardized loading (SE) |
| --- | --- | --- | --- |
| Social support | |  |  |
|  | Item 1 | 1 | 0.81 (0.01) |
|  | Item 2 | 0.98 (0.03) | 0.77 (0.02) |
|  | Item 3 | 0.73 (0.04) | 0.63 (0.03) |
|  | Item 4 | 1.00 (0.03) | 0.78 (0.02) |
|  | Item 5 | 1.04 (0.03) | 0.86 (0.01) |
|  | Item 6 | 0.88 (0.04) | 0.76 (0.02) |
|  | Item 7 | 0.89 (0.04) | 0.74 (0.02) |
|  | Item 8 | 0.80 (0.04) | 0.67 (0.03) |
| Mental distress | |  |  |
|  | Item 1 | 1 | 0.86 (0.01) |
|  | Item 2 | 0.98 (0.03) | 0.92 (0.01) |
|  | Item 3 | 0.99 (0.03) | 0.92 (0.01) |
|  | Item 4 | 1.05 (0.03) | 0.92 (0.01) |
| Self-efficacy | |  |  |
|  | Item 1 | 1 | 0.89 (0.01) |
|  | Item 2 | 0.76 (0.03) | 0.85 (0.01) |
|  | Item 3 | 0.77 (0.02) | 0.87 (0.01) |
|  | Item 4 | 0.70 (0.03) | 0.85 (0.01) |
|  | Item 5 | 0.78 (0.02) | 0.87 (0.01) |
|  | Item 6 | 0.77 (0.03) | 0.88 (0.01) |
|  | Item 7 | 0.75 (0.03) | 0.82 (0.02) |
|  | Item 8 | 0.72 (0.03) | 0.86 (0.01) |
|  | Item 9 | 0.81 (0.03) | 0.89 (0.01) |
|  | Item 10 | 0.82 (0.03) | 0.89 (0.01) |
|  | Item 11 | 0.73 (0.03) | 0.84 (0.02) |
|  | Item 12 | 0.76 (0.03) | 0.87 (0.01) |
|  | Item 13 | 0.83 (0.03) | 0.86 (0.01) |
